# Multistage fuzzy comprehensive evaluation of landslide hazards based on a cloud model

**DOI:** 10.1371/journal.pone.0224312

**Published:** 2019-11-05

**Authors:** Xuelin Yao, Hongwei Deng, Ti Zhang, Yaguang Qin

**Affiliations:** School of Resource and Safety Engineering, Central South University, Changsha, China; Shandong University of Science and Technology, CHINA

## Abstract

To accurately study the risk assessment of landslide disasters, firstly, the environmental conditions of induced landslide disasters are regarded as a fuzzy system, and the landslide risk factors in the multi-level analysis system are constructed to build a multi-level fuzzy evaluation index system. Then, the cloud model theory is introduced to improve the importance scale and membership degree involved in the evaluation process, and the multi-level fuzzy comprehensive evaluation method of landslide risk improved by a cloud model is proposed. Thus, a multi-level fuzzy evaluation cloud model for evaluating landslide risk is established. Finally, using the improved cloud model method, a multistage fuzzy comprehensive evaluation of landslide risk is conducted for the K112+210~K112 +630 section of the Long Chuan to Huaiji Highway Project in Guangdong Province. The results show that the improved cloud model can solve the problem of uncertainty in the process of landslide preparation and occurrence, greatly improve the effectiveness of landslide evaluation results, and provide an effective reference for landslide disaster prevention.

## Introduction

A landslide is a serious natural disaster, that can do great harm to the development of human society and economic construction. Seventy percent of the areas in China are mountainous areas, especially in the southwest hilly and mountainous areas of China. The topographic and geomorphological features include numerous mountains, steep mountains, loose soil structure, easily stagnant water, and valleys and rivers all over the mountains, and the mountains cut each other to form a large number of slopes and cutting surfaces with enough sliding space. There are a wide range of basic conditions for the occurrence of landslides in China, so the frequency and density of landslides are high; in addition, China is one of the countries that is most seriously affected by this type of disaster in the world.

In June 2017, a very large landslide disaster occurred in Xinmo Village, Diexi Town, Maoxian County, Aba Prefecture, Sichuan Province. In this disaster, more than 40 farmhouses and more than 100 people were buried, and 2 kilometres of rivers were blocked, resulting in very large losses of life and property. In July 2013, a landslide disaster occurred in Zhongxing Town, Dujiangyan City, Sichuan Province. When the disaster occurred, it rained heavily, and the high landslide speed was fast, forming a landslide body with a width of 300 m, a longitudinal length of 150 m and a volume of more than 1.5 million cubic metres. The disaster affected 2.094 million people in Sichuan and caused great losses. With the continuous development of social economy, human beings develop land on a larger scale. In addition to natural landslides, human beings employ unreasonable methods in the process of land development, resulting in increasingly many serious landslide disasters that endanger human safety. Briefly, hundreds of millions of losses caused by landslide disasters in China every year have a serious social impact, and have caused significant losses of lives and property. Therefore, it is very meaningful to study risk assessment and analysis of landslide disasters.

At home and abroad, landslide risk research has been carried out since the 1960s, with a large number of studies. The evaluation results are increasingly accurate, the methods are increasingly abundant, and a mature theory and evaluation technology has gradually been developed and applied.

In Van Dijke J.J et al (1990) [1], through field investigation and data analysis, a large number of geological hazard data were counted and collected, on the basis of which a risk rating model was established and a geological hazard risk assessment was performed.

Gupta R P et al (1990) [2], analysed the risk coefficient of a landslide in the Ramgenga catchment, and combined with geographic information system, the risk coefficient was used to measure the risk of landslide.

In Guzzetti et al (2005) [3], based on the landslide risk of a watershed in Italy, the landslides were classified according to different time periods and selected as the basic evaluation units, and the time probability of landslide occurrence in different time periods was analysed.

A. Uormeihy et al (2000) [4–5], evaluated the landslide risk in Iran, selected the grid unit as the basic evaluation unit; established an index system based on the indexes of topographic slope, stratum lithology, geological structure, river erosion and land use; completed a landslide risk assessment in this area; and generated relevant maps of the landslide risk.

Miles and Keefer (2009) [6] took seismic landslides as the research object, proposed a technical framework for landslide risk assessment, developed a camel model of bedrock logical regression, and applied the model to an example.

In recent years, scientists in various countries generally use topography, geomorphology, strata, lithology, geological structure and other environmental factors in landslide risk zoning.

Based on the characteristics of geological structure, stratum structure, slope pattern, slope geometry and climatic conditions, Al-Homoud A S and Masanat Y [7] studied the landslides along the Jordan expressway that occurred in the past 25 years. According to the relevant experience, each factor was weighted, and the stability of the landslides was evaluated.

Based on geographic information systems (GIS) techniques, Barredo J et al [8–9] studied the movement of the Gran Canaria Island block in Triajana Basin, Spain, using a direct method and an indirect method. The direct method involves obtaining very detailed topographic map data, using unique coding polygons and employing an expert system to evaluate the type and degree of landslide risk. The indirect method is similar to the determination index method. The slope angle, the landslide activity, the development stage of the landslide, the material composition of the landslide, the water storage of the landslide and the change of land use are selected as parameters to combine these parameters. It is applied to landslide risk assessment.

In 1989, Qiao Jianping et al [10] selected 12 evaluation indexes from the signs of slope deformation, internal conditions and trigger factors, which were qualitative and semi-quantitative, and assigned 6 grades of discriminant index. The method of direct superposition of discriminant factor indexes was used to establish, a semi-quantitative evaluation model for slope risk assessment, and the risk degree was divided into six grades and applied to Muli County, as a practical application.

In 1999, Zheng Qianqiang [11] conducted a risk assessment of a typical landslide in Jiangxi Province, which was a semi-quantitative assessment. The selected evaluation factors were the landslide front, back wall, parent rock lithology, residual soil thickness and artificial cut slope. Qualitative assignment and an expert scoring method were used to determine the weight of each factor. Finally, through the fuzzy comprehensive evaluation method, the risk degree of landslide was calculated, according to the principle of the maximum membership degree.

In 2001, Wang Chenghua [12–13] analysed the internal and external conditions and deformation characteristics of landslides; established a three-level evaluation index system for quantifying the high-speed landslide risk; and according to expert experience, assigned each index with an action index, and performed three-level division and evaluation of the landslide risk degree.

In 2004, Fan Xiaoyi and others [14] applied an analytic hierarchy process (AHP), constructed a comparison matrix with the help of expert opinion, took its eigenvector as the weight value for the single landslide evaluation factor, sorted the importance of the evaluation factors, and evaluated the risk degree of the Baota landslide.

Tang Hongmei et al [15], used an Analytic hierarchy process (AHP) to perform a risk assessment of Wujiawan landslide from a semi-quantitative point of view.

In 2016, Feng Hangjian [16] comprehensively considered the spatial probability, time frequency and landslide strength of landslides, carried out a landslide risk assessment in Chun'an County, and proposed some suggestions for disaster prevention and mitigation of landslide disasters in this area.

With the frequent occurrence of geological disasters and the rapid development of landslide science, single qualitative evaluations of landslide risk have lacked innovation. Quantitative study of single landslides is key for studying landslide risk.

At present, the most commonly used method for analysing single landslide is landslide stability analysis. In the early stage, it was based on the Swedish arc method and slice method, and it was later improved by Janbu and Bishop et al [17].Specifically, it was changed to calculate the safety factor K via the limit equilibrium method, and judge the stable state of the landslide according to the K value. The stability of a single landslide is obtained via the limit equilibrium method, and the risk degree of the single landslide is analysed. The C value and P value are needed in the process of calculation, but because of the uncertainty in the C value and P value, the objectivity of the result will be seriously affected. In addition, in the stable-state division, a stability coefficient greater than 1 indicates a stable state, whereas less than 1 indicates an unstable state, this lack of a continuous measure of the risk indirectly affects the evaluation value of the landslide risk degree, so the method cannot meet the needs of single-landslide quantitative analysis.

Generally, the risk assessment of geological disasters such as landslides is a function operation based on the evaluation index and evaluation model, and the result is the danger or risk degree. The evaluation indexes are generally historical factors, the point density, environmental factors, terrain slope, inducing factors, rainfall, earthquakes and so on.

From the engineering point of view, regional landslide risk assessment requires a lot of manpower, financial and material resources, which is not conducive to the timely evaluation of individual landslides and dangerous slopes. Moreover, the traditional method cannot address uncertainty in the evaluation index. Therefore, this paper proposes an effective risk assessment method for a single landslide, and optimizes the evaluation method according to the subjectivity and randomness of the evaluation index; the method can accurately evaluate the risk degree of a single landslide disaster and obtain its harm degree. It is of great significance to formulate disaster prevention and mitigation measures.

Therefore, this paper attempts to address the overall combination of environmental conditions of induced landslide as a fuzzy system, through a top-down decomposition of various induced landslide hazard sources to construct the landslide risk assessment index system. According to the results of the constructed system, combined with the cloud model theory, the randomness in the process of weight and membership degree of each index factor is improved and determined, such that a multi-level fuzzy comprehensive evaluation of landslide risk can be carried out more reasonably. A landslide on the TJ15 section of the Longchuan to Huaiji Highway Project in Guangdong Province is selected as the research object, and the research results are applied to the evaluation of the research object, which makes the evaluation results more in line with reality and provides a reliable basis for the prevention and treatment of landslides.

## Landslide hazard evaluation index system

**Fig 1:**
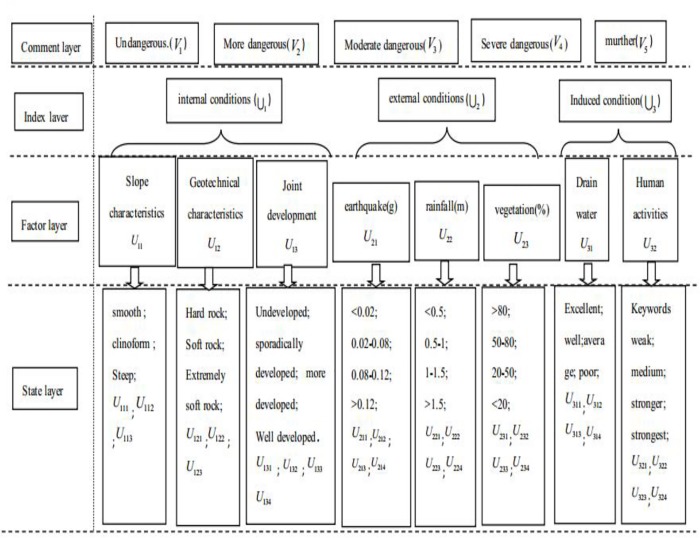
Multilevel index system for landslide hazard assessment.

To evaluate the risk of landslides, it is necessary to reveal the corresponding hazard sources. The related research results suggest [18] that a landslide must have certain conditions, including internal conditions (U_1_), external conditions (U_2_) and induced conditions (U_3_) to form. The internal conditions (U_1_) are mainly related to the slope (U_11_), geotechnical characteristics (U_12_), and factors such as the joint characteristics (U_13_). The external conditions (U_2_) mainly include earthquake characteristics (U_21_), rainfall (U_22_) and vegetation (U_23_), and the induced conditions (U_3_) include drainage (U_31_) and human activities (U_32_) [19]. These factors can be further divided into different states according to their specific characteristics. According to this principle, an AHP is used to systematically identify the landslide risk. Different landslide hazard grades are used as evaluation layers, and these grades are non-dangerous (V_1_), relatively dangerous (V_2_), moderately dangerous (V_3_), severely dangerous (V_4_) and extremely dangerous (V_5_) [20]. The index layer encompasses the inner, external condition and induced conditions of the landslide, and the index layer is decomposed into a factor layer and state layer, which includes the comment layer and the index layer. The evaluation index system of landslide risk [19–21] is shown in Fig 1.

## Landslide risk assessment model

### Multilevel fuzzy comprehensive evaluation model

Multilevel fuzzy comprehensive evaluation [22–24] is a widely used multifactor and multiobjective decision-making approach. This method integrates the advantages of the analytic hierarchy process and fuzzy comprehensive evaluation. These advantages are mainly reflected in the multilevel structure of the evaluation index system. The index weight of each layer is determined by an AHP, and a comprehensive evaluation of the results is performed at different levels. The overall evaluation results are given at the end. The development and occurrence processes of landslides form a complex fuzzy system. Based on the risk evaluation index system discussed above, a multistage fuzzy comprehensive evaluation method can be used to comprehensively evaluate the risk.

Let U = {U_1_ (internal conditions), U_2_ (external conditions), U_3_ (induced conditions)}. Among these variables, U_1_ = {slope characteristics U_11_; geotechnical characteristics U_12_; joint development U_13_;}, U_2_ = {earthquake (g) U_21_; rainfall (m) U_22_; vegetation (%) U_23_}, and U_3_ = {drainage U_31_; human activities U_32_}.

Let V be a set of landslide risk grades. In Fig 1, V = {V_1_ (not dangerous), V_2_ (more dangerous), V_3_ (moderate danger), V_4_ (severe danger), V_5_ (extremely dangerous)}.

Based on the established multilevel index system of landslide risk assessment, as long as the weight value and membership degree of each layer and node are determined, multilevel fuzzy mapping can be adopted. The comprehensive evaluation of landslides is performed with a specific combination of environmental factors to determine the induced landslide hazard grade. The composition algorithm for fuzzy comprehensive evaluation [25] is as follows:

S=W•R

where W is the weight vector, which is composed of the weight of each landslide factor; R is the subordinate degree of the subordinate child nodes for a given parent node; and S is the comprehensive evaluation result matrix at this level.

The results of the comprehensive evaluation corresponding to the evaluation layer form the final evaluation result matrix of the multilevel fuzzy comprehensive evaluation model. According to the principle of the maximum membership degree, the risk level corresponding to the element with the largest membership degree is the corresponding risk level. This level is the final evaluation result of the multilevel fuzzy comprehensive evaluation model.

In the process of multistage fuzzy comprehensive evaluation of landslide risk, the fuzziness of complex landslide systems is considered, but the randomness and discreteness of the system are neglected. This limitation can be improved by employing a cloud model.

### Improved multilevel fuzzy comprehensive evaluation with a cloud model

The first cloud model was a qualitative and quantitative interconversion model proposed by Deyi [26] in the 1990s. Uncertainty can be represented by different factors, such as fuzziness, randomness and discreteness. Based on traditional probability theory and fuzzy mathematics, the cloud model quantifies the expectation (Ex), entropy (En) and hyperentropy (He). Additionally, fuzziness, randomness and discreteness are organically combined to provide a natural transformation between uncertain language and quantitative value [27–28]. A cloud model can be used to improve the multistage fuzzy comprehensive evaluation model by considering the fuzziness in a landslide evaluation system. At the same time, this approach encompasses the randomness and discreteness of the system; thus, under uncertainty, a comprehensive evaluation of landslide hazards can be performed.

In a multilevel fuzzy comprehensive evaluation model of landslides, the randomness and discreteness of the system are mainly reflected by subjective opinions. First, to avoid the influence of the personal experience of experts and other subjective factors on the evaluation results, when determining the matrix of landslide factors, it is necessary to use the method of group decision making to make the decision. However, the traditional aggregation method is only a simple algebraic operation based on the expert score, but each expert score has inherent fuzziness, randomness and discreteness. Additionally, only simple algebraic operations are applied, resulting in approximate results. Moreover, it is difficult to use an accurate numerical value to objectively represent the membership degree of the landslide risk grade. The existing methods usually use subjective values or empirical formulas to obtain the membership degree. This process creates uncertainty associated with the membership levels.

**Fig 2:**
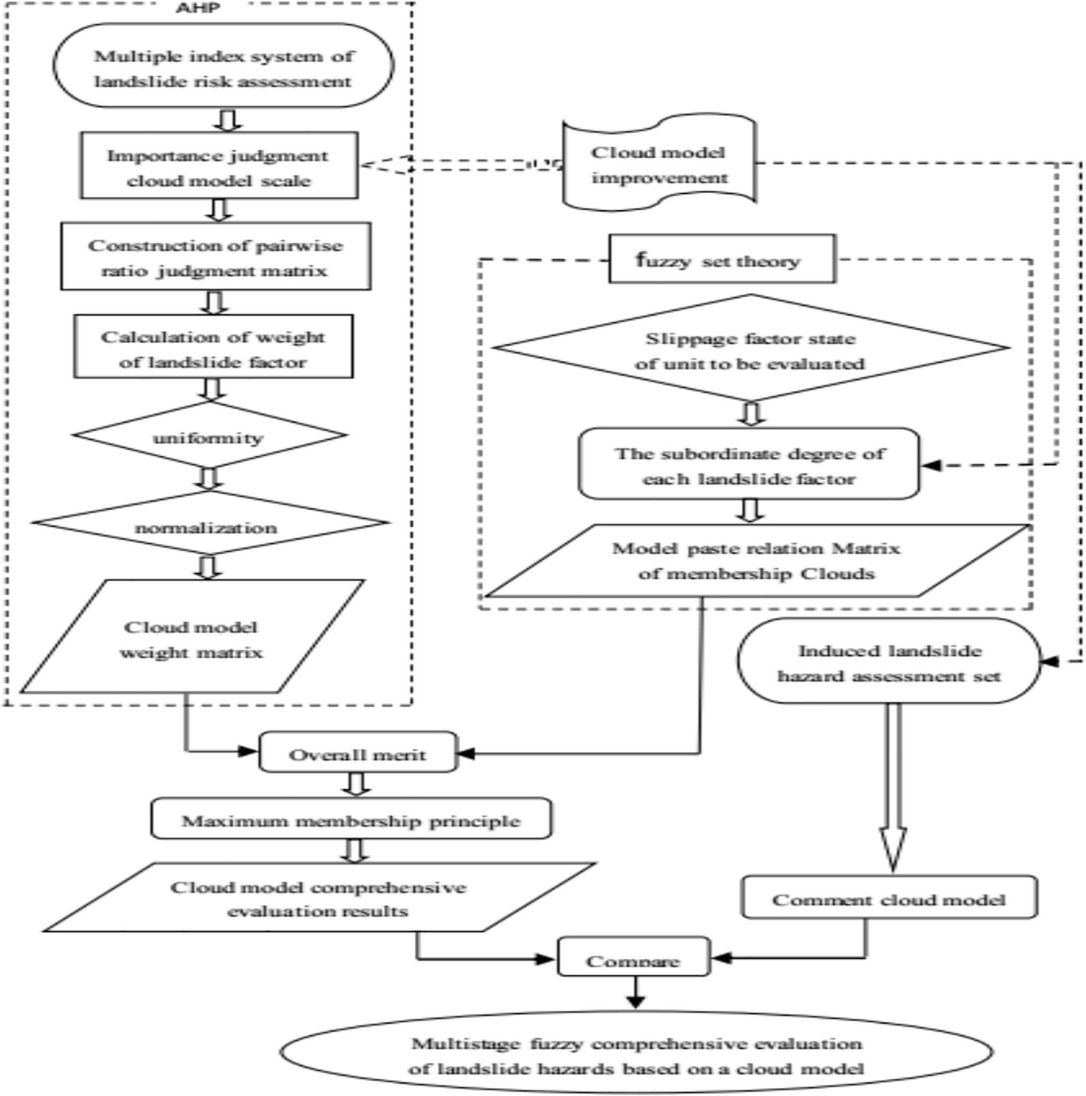
Flowchart of the improved multistage fuzzy evaluation model for landslide hazards based on a cloud model.

Therefore, this paper uses an improved multilevel fuzzy comprehensive evaluation method with a cloud model to construct a landslide risk model. The key points of this model include the use of the cloud model, the scale of the cloud model and the membership function of the cloud model. Each cloud model is characterized by its corresponding Ex, En and He values, which are represented as U (Ex, En, He). Ex reflects the centre of gravity of the cloud droplet, which indicates the landslide hazard grade, landslide factor weight and subordinate degree. En encompasses the fuzziness and randomness of cloud droplets, as well as the possible ranges of landslide risk grades, landslide factor weights and membership degrees. He is the entropy. This variable is used to describe the thickness of the cloud and mainly reflects the dispersion degree of cloud droplets, indicating the deviations in the degree of landslide danger, the landslide factor weights and the subordinate degree from the average values. The flowchart of the multilevel fuzzy comprehensive landslide evaluation model with a cloud submodel is shown in Fig 2.

The advantages of this model are as follows: (1) unlike the comment layer determined by the traditional set theory method, the evaluation cloud model obscures the boundary of the risk level, which is more suitable for the language habits of humans. Thus, the subjective uncertainty in the process of comparing the evaluation results is reduced. (2) Considering the difficulty of selecting the importance scale in the pairwise comparison judgement matrix, using the cloud model scale to describe the linguistic variables in expert scoring can more accurately describe the relative importance among factors. (3) Unlike the traditional empirical formula method, the membership degree of each evaluation factor corresponding to the risk level is determined by the cloud model. The cloud model of the membership degree function is constructed, and the relationship between the landslide factor and risk level is expressed by three numerical features: Ex, En, and He. Then, one-to-many mapping between qualitative and quantitative factors is performed.

## Application example and analysis results

### Case data

To verify the improved multistage fuzzy comprehensive evaluation model of landslide risk, this paper investigates a landslide body in the TJ15 section of the Long Chuan to Huaiji Highway Project in Guangdong Province. This body is located on the right side of a deep cutting excavation from section K112+210 ~ K112+630 of the Long Chuan to Huaiji Highway. The direction of the route is at approximately 259°.

**Fig 3:**
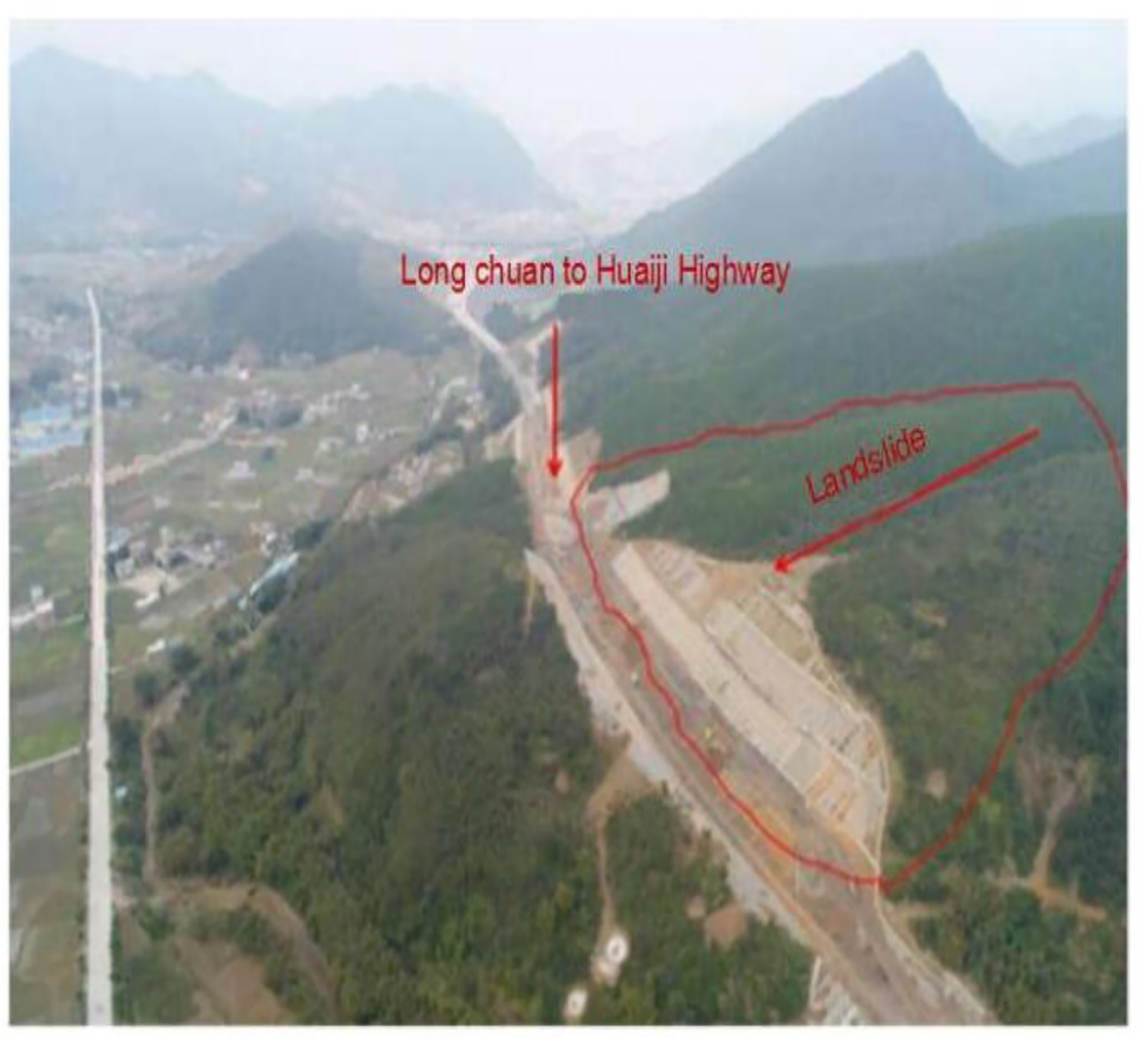
P lane schematic diagram of relative relationship between landslide and line.

The total length of the cutting is approximately 420 m, and the maximum excavation depth of the middle line is 25.847 m, which is located at K112+460. After excavation, a slope of approximately 20 m will be formed on the left side and 40 m on the right side. The relationship between the landslide and the location of the line is shown in Fig 3.

A practical engineering test is conducted. The landslide is a giant ancient landslide project, and the induced landslide has become the research focus of scholars worldwide. Because the landslide spans many geomorphological units, there are considerable differences in the environmental conditions of the landslide in different regions, so the hazard magnitude of the complex landslide is determined by many factors and spatially varies. According to the uncertain characteristics of these factors, the subdivision method is applied to the landslide. According to the environmental conditions of the landslide, the landslide body is divided into several evaluation units, and landslide risk assessment is conducted for each element [26]. Taking any element as an application example, the landslide environment conditions are reported in Table 1, and the evaluated unit is in a non-dangerous state. Similarly, other units can be evaluated for landslide risk.

**Table 1:** State data for the induced landslide environmental condition factors in the selected computational unit.

Landslide factor	*U*_11_	*U*_12_	*U*_13_	*U*_21_	*U*_22_	*U*_23_	*U*_31_	*U*_32_
Status	*U*_111_	*U*_121_	*U*_131_	*U*_211_	*U*_221_	*U*_231_	*U*_311_	*U*_321_

After obtaining the environmental condition data for the landslide, the improved square method based on the cloud model proposed in this paper can be used to calculate the reservoir-induced seismic risk of the evaluation unit according to a multistage fuzzy comprehensive evaluation. The normal cloud model is the most basic cloud model, and probability theory suggests that the expected curves of many social science and natural science phenomena are similar to the normal distribution. Therefore, all cloud models in this paper are constructed as normal cloud models.

### Evaluation cloud model for landslide risk

According to the multilevel index system of landslide risk evaluation shown in Fig 1, sets V denote non-dangerous (V_1_), relatively dangerous (V_2_), moderately dangerous (V_3_), severely dangerous (V_4_) and extremely dangerous (V_5_) conditions respectively. These characteristics can be represented by cloud models with the corresponding Ex, En and He values.

According to the evaluation ranges of V_1_-_5_, the normal cloud model is used to express the corresponding Ex values. En and He can be calculated with the following formulas:

$$\left\{\begin{array}{c} E_{x}=(I_{min}+I_{max})/2\\ E_{n}=(I_{max}‐I_{min})/6\\ H_{e}=k \end{array}\right\}$$

where *I*_max_ and *I*_min_ are the maximum and minimum values, respectively, and K is a constant reflecting the fuzzy threshold of the comment layer, which can be adjusted according to the specific comment. In this paper, k is taken to be 1. The results are reported in Table 2.

**Table 2:** The number of standard cloud model features corresponding to the comment set.

Comment set	Representation		
	*E*_*x*_	*E*_*n*_	*H*_*e*_
**Non-dangerous *V***_**1**_	10	3.3333	1
**Relatively dangerous *V***_**2**_	30	3.3333	1
**Moderately dangerous *V***_**3**_	50	3.3333	1
**Severely dangerous *V***_**4**_	70	3.3333	1
**Extremely dangerous *V***_**5**_	90	3.3333	1

**Fig 4:**
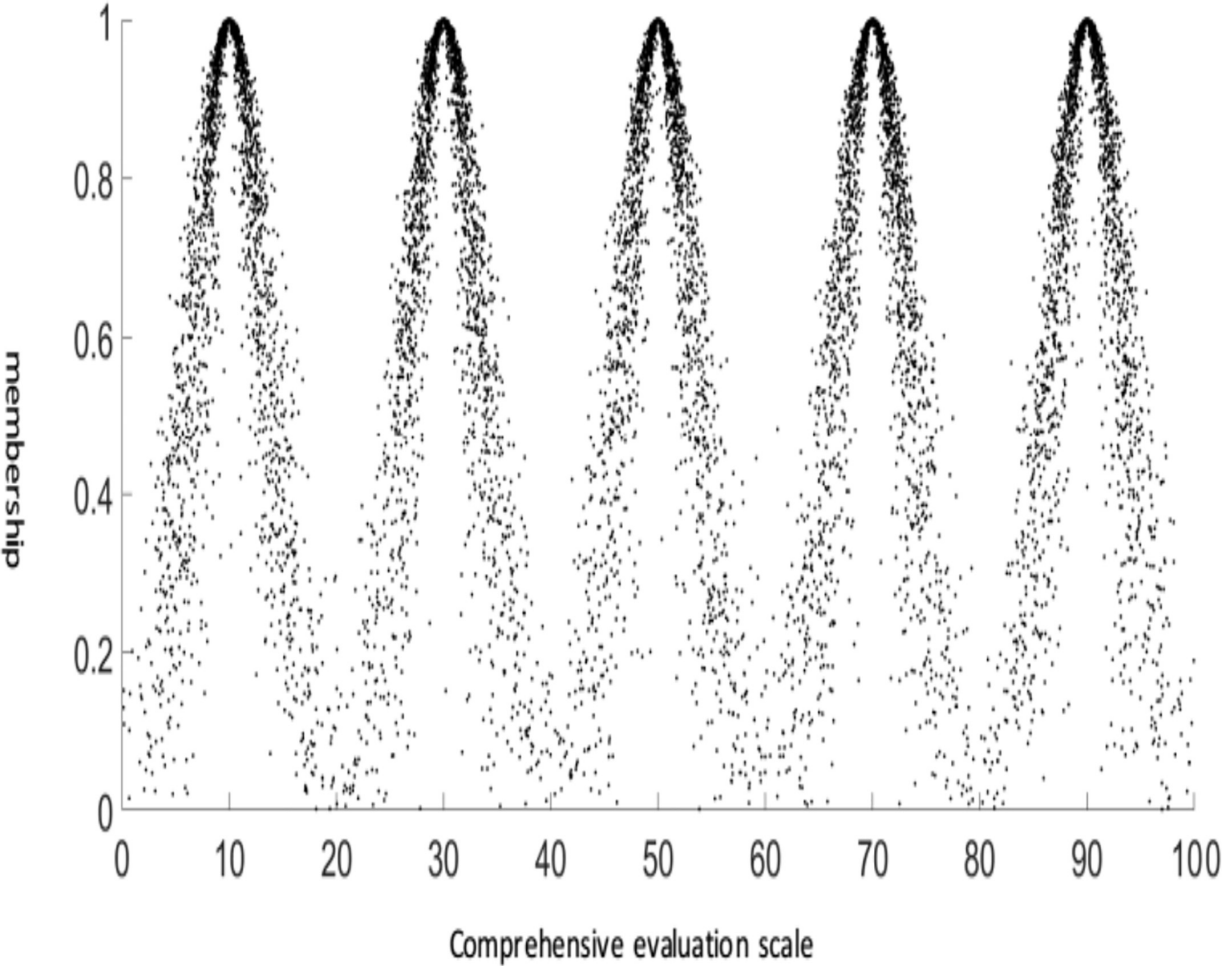
Evaluation of landslide risk assessment based on the cloud model.

As shown in Fig 4, five cloud models can be used to represent the five levels of risk assessment for induced landslides: non-dangerous (V_1_), relatively dangerous (V_2_), moderately dangerous (V_3_), severely dangerous (V_4_) and extremely dangerous (V_5_). Using the cloud model for reference in landslide risk assessment, the target layer of induced landslide risk assessment determined by the cloud transformation method not only blurs the risk boundaries but also fully considers the randomness and discretization of the cloud model. These abilities are based on the advantages of cloud model theory. The standard comment cloud model was generated with the MATLAB software package.

### Scale of the cloud model for landslide-induced factors

One of the important steps in calculating the weights of index factors using an AHP [24, 29] is to construct a pairwise matrix of moments, and the key is selecting the appropriate scaling method. In the Satty [23, 29] scale of the classical AHP, experts are required to determine the relative importance of two factors based on a number between 1 and 9. In the state analysis of the induced slope factor, the state of the landslide factor can be simplified by dividing the evaluation unit into subunits, and the state of the landslide factor can be reduced to a certain degree. Regarding these landslide factors, which rely mainly on language to distinguish their state, comparisons of their importance are often greatly influenced by individual expert experience and subjective factors. In this paper, we construct a pairwise comparison judgement matrix of induced landslide factors based on the cloud model scale. The scaling criteria of the cloud model [29] are listed in Table 3.

**Table 3:** Scaling criteria for cloud models.

Scale	Meaning
1 3331 56.330 10 1	I is as important as j 0.045
3	I is slightly more important than j
5	I is more important than j
7	I is much more important than j
9	I is extremely much more important than j
2, 4, 6, 8	Represents the intermediate value of the above adjacent judgement
count backward	If the ratio of the importance of factor I to that of factor j is *B*_*ij*_, then the ratio of the importance of factor j to that of factor I is *B*_*ji*_ = 1/*B*_*ij*_

After the importance scale criteria are established, the importance of each landslide factor can be compared, the judgement matrix can be constructed, and the weight of each factor can be calculated by the judgement matrix. The most commonly used methods for this calculation are the square root method and the eigenvalue method. The sum product method and the least square weight method are used to construct the judgement matrix from the scale of the cloud model, and the square root method is used to calculate the weight of each landslide factor. By calculating the elements of each row in the pairwise comparison judgement matrix, the formulas for calculating Ex, En, and He of the weighted cloud can be obtained as follows.

$$\begin{array}{l} Ex=\frac{\sum_{i=1}^{n}Ex_{i}En_{i}v_{i}}{\sum_{i=1}^{n}En_{i}v_{i}}\\ En=\sum_{i=1}^{n}En_{i}v_{i}\\ He=\frac{\sum_{i=1}^{n}He_{i}En_{i}v_{i}}{\sum_{i=1}^{n}En_{i}v_{i}} \end{array}$$

The corresponding Ex, En, and He data are reported in Table 4.

**Table 4:** Characteristic parameters of the weighted cloud model for each landslide factor.

Index	Cloud model feature number	Index	Cloud model feature number
U11	(81.766, 5.013, 1.363)	U12	(39.152, 3.333, 1)
U13	(48.32, 3.333, 1)	U23	(63.187, 5.525, 1.756)
U21	(72.75, 3.333, 1)	U22	(45, 3.333, 1)
U31	(37.017, 5.113, 1.367)	U32	(44.746, 6.072, 1.680)

Compared with a traditional AHP, the weight of each landslide factor is calculated using the judgement matrix at the cloud model scale. The importance scales are compared, and En and He are calculated. A more objective description of the fuzziness and discreteness of the evaluation language is given. Similar to a traditional AHP, it is necessary to check the consistency of the judgement matrix, that is, to check the expected values of the elements in the cloud model judgement matrix.

### Membership cloud model of landslide factors

According to the principle of the reverse membership cloud generator, the membership function for each landslide factor can be established via what is essentially a statistical analysis process.

$$\begin{array}{l} Ex=\frac{1}{p}\sum_{k=1}^{p}x_{k}\\ S^{2}=\frac{1}{p‐1}\sum_{k=1}^{p}{\left(x_{k}‐Ex\right)}^{2}\\ En=\sqrt{\frac{\pi }{2}}\times \frac{1}{p}\sum_{k=1}^{p}\left|x_{k}‐Ex\right|\\ He=\sqrt{S^{2}‐En^{2}} \end{array}$$

The Ex, En and He values of the cloud model are determined, and the corresponding membership cloud model is obtained. For an arbitrary evaluation unit, eight corresponding cloud membership functions can be extracted from the membership function library as long as eight landslide factors are known. The traditional membership function is usually a definite curve, which makes the determination of the membership degree a conversion from fixed to quantitative values. The fuzziness and randomness of risk factors can be combined to perform one-to-many mapping between qualitative and quantitative factors using the membership functions from the cloud model. The mathematical characteristics of the membership degree are represented by the three numerical eigenvalues of Ex, En, and He, and the randomness and discreteness of the relationships between the landslide factors and the membership degree of the risk level are fully considered. In this case, Ex represents the expected value of risk, En denotes the degree of dispersion of the membership degree relative to the expected value, and He represents the degree of deviation of the true membership degree from the expected value.

### Cloud model for the comprehensive evaluation of landslide risk

**Fig 5:**
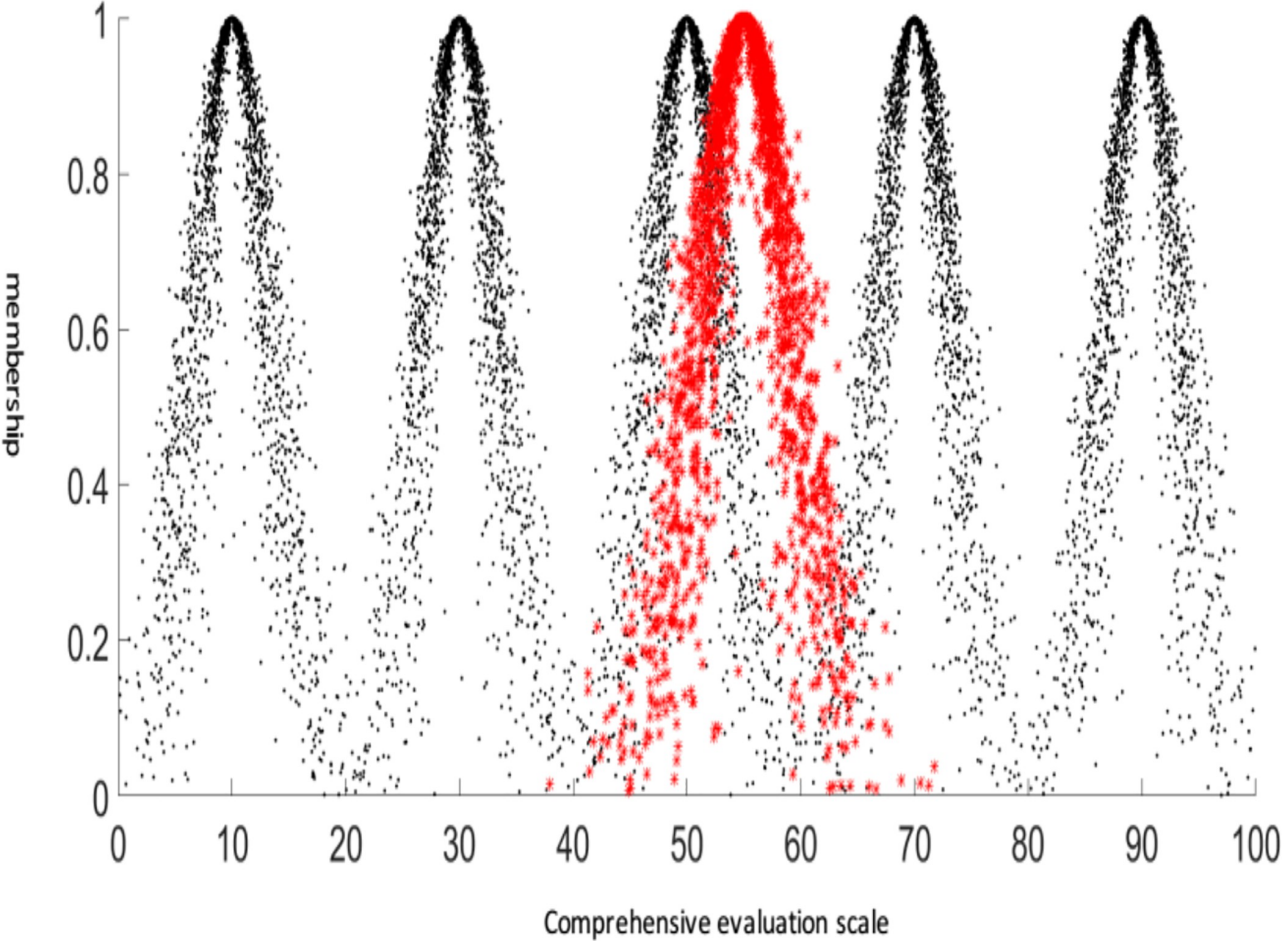
Comparison of the cloud model and the risk assessment cloud model.

The traditional multilevel fuzzy comprehensive evaluation method typically uses the principle of the maximum membership degree or the weighted average method to make comprehensive decisions. The former may lead to a large judgement deviation because only the maximum value of the membership degree is considered. Because there are many subjective factors associated with the latter approach, it is difficult to achieve satisfactory results. Therefore, this paper combines the cloud model with the multilevel fuzzy comprehensive evaluation method based on the established landslide risk assessment cloud model, the landslide factor weight cloud model and the subordinate degree cloud model. According to the basic operation rules of the cloud model, the final landslide risk evaluation result for an example unit is evaluated by the cloud model, and a certain expectation is adopted. The cloud models of entropy and hyperentropy are used to describe the comprehensive evaluation results for landslide risk. The weighted cloud model of the slope elements and the membership degree cloud model corresponding to the 8 landslide factor states are obtained by weighted average operations. Finally, the improved cloud model for the multistage fuzzy comprehensive evaluation of landslide risk is obtained as Cloud Ex 55.051, En 4.091, and He 1.248. This cloud model is represented by the red curve in Fig 5.

The comprehensive evaluation results of the cloud model and risk assessment cloud model were assessed for each level of risk (V_1_, V_2_, V_3_, V_4_ and V_5_). The cloud results (red) lie between "moderate danger V_3_" and "severe danger V_4_", and Ex is 55.051. The results indicate that a landslide in this unit is most likely to be classified in the moderate danger or severe danger categories, but a landslide of moderate risk will most likely to be induced. The En and He values of the cloud are 4.091 and 1.248, respectively. These values are small, so the cloud distribution of the evaluation results is relatively concentrated. This finding shows that the possible landslide grade induced by this unit has a centre value of 55.0511, and slightly higher- or lower-grade landslides could be induced within a small range.

## Conclusions

By treating the integrated environmental conditions of landslides as a complex fuzzy system with uncertainty, the main risk sources of landslides can be analysed and determined by risk identification. In this study, the traditional multistage fuzzy comprehensive evaluation method of landslides is improved using a cloud model. Through the scale and weight cloud models, a membership function is used to determine the membership degree of each landslide factor, and the uncertainty in the evaluation process is avoided to the greatest extent possible. The result of comprehensive risk evaluation is a cloud model with three numerical characteristics: Ex, En, and He. The cloud model is used to describe the comprehensive evaluation results of landslide risk. The central value, model fuzziness and randomness of the evaluation results are considered, and the robustness of the risk assessment is greatly improved compared to the traditional approach. A new, simple and effective method is provided for quantitative analyses of the uncertainty in the landslide development and occurrence processes. This paper combined a cloud model with a multilevel fuzzy comprehensive evaluation method to evaluate landslide risk, and the following conclusions were obtained.

1. The index system of landslide risk assessment, including 3 first-grade indexes and 8 second-grade indexes, was constructed, and the weights and evaluation criteria for each index were determined. Cloud model theory was applied in the risk assessment system. A model of landslide hazard assessment based on a cloud model was established.
2. Based on calculations for an actual landslide, the comprehensive cloud number characteristics of landslide risk assessment are U = (55.051, 4.091, 1.248). From the cloud droplet diagram and calculation results, the level of risk in the landslide risk assessment is "moderate danger". The evaluation results are in line with the actual situation in the area.
3. Using a cloud model is a novel research method in the study of landslide hazard, which is based on consulting a large number of published studies and books. Therefore, how to excavate the value of cloud model in landslide risk assessment is a problem worthy of further study.
4. The application of cloud model theory to landslide risk assessment expands the application of this theory in the field of landslide risk assessment, how to select more practical evaluation indexes, and how to more objectively and accurately define the area of each stability grade. It is a problem that can be further discussed.
